# ADHD help-seeking attitudes of Asian Americans

**DOI:** 10.3389/frcha.2024.1491978

**Published:** 2025-01-23

**Authors:** Monisha Chawla, Jennalyn Vandenheuvel, Shannon L. Sibbald

**Affiliations:** Western Centre for Public Health and Family Medicine, Schulich Interfaculty Program in Public Health, Western University, London, ON, Canada

**Keywords:** help-seeking, ADHD, Asian Americans, model minority myth, risk factors, protective factors

## Introduction

Attention-deficit/hyperactivity disorder (ADHD) is a commonly diagnosed disorder among children, with a global incidence of 7.2% in those under 18 years old (1). Despite extensive research, there remain significant variations in the diagnosis and management of ADHD across different cultures (1). Although previous studies have highlighted disparities in ADHD diagnosis and treatment for Hispanic and African American populations, there is a notable gap in understanding the experiences of Asian populations. One review found that Asian children had the lowest incidence of ADHD across ethnic groups, but this may be due to differences in symptom presentation and cultural practices (2). This is critical to note, as Asian Americans are one of the fastest-growing ethnic groups in America, but remain largely overlooked in federal behavioral health policies (3). Furthermore, limited research has shown that Asian children with ADHD may be less likely to receive treatment than their peers (4). While Asian American communities have been underrepresented in ADHD research, there have been studies examining their attitudes towards mental health help-seeking behaviors for other conditions. It is noteworthy that the term Asian American refers to a diverse population, comprising Vietnamese, Chinese, Korean, Asian Indian, and Filipino groups (5). These groups exhibit a wide range of cultures and beliefs, making categorizing them under one label challenging. However, this is often how they are portrayed in literature.

This paper seeks to provide an overview of the social and cultural factors that affect Asian Americans help-seeking behaviors and present suggestions for the adaptation of a help-seeking model for this population. The proposed adjustments will be made to the model from Eiraldi et al. (6) (Appendix A) that highlights the stages in the parental help-seeking pathway for ethnic minority children with ADHD. This model is adapted from Cauce et al.'s, 2002 stages of help-seeking model to include factors that impact ethnic minority help seeking decisions. The original model has four stages of help-seeking behavior which include: problem recognition, decision to seek help, service selection, and service utilization (7). Although this model is dated, a recent qualitative study on Asian American help-seeking behaviors found that themes regarding behaviors largely correlated with Cauce's stages of help-seeking (8), indicating that the model is still relevant. As Asian American populations continue to grow and ADHD diagnoses become more common, it is crucial to establish a culturally sensitive help-seeking approach. This model may not only identify factors that hinder or enable diagnosis and access to services but also inform the creation of policies and programs to reduce treatment disparities for ethnic minority groups.

## Factors that influence help-seeking behaviors

There are several underlying assumptions around Cause et al.'s help-seeking model that impacts one's ability to receive help. First is the assumption that individuals must recognize their health concerns before seeking help (6, 7). Once this recognition occurs, individuals must weigh the pros and cons of various treatment options and decide if they are willing to pursue help. Finally, they select and participate in the service they believe best meets their needs (6, 7). This model is very similar to the Health Belief Model, where an individual decides to seek care based on their perceptions of susceptibility to an illness, severity of the health concern, barriers to seeking help, and benefits of seeking care (9). It is imperative to comprehend the various stages and underlying beliefs of help-seeking models before exploring the specific Asian American cultural factors that shape these stages.

### Model minority myth

The “model minority myth” is a phenomenon that is unique to Asian populations. It suggests that Asian populations are viewed as a modern-day American success story, as they are perceived to have attained relatively high educational and occupational statuses without many difficulties adjusting (10). This perception often leads to the homogenization of a diverse group of individuals, resulting in an oversimplification and lack of acknowledgement of the challenges faced (10, 11). The myth suggests that Asian populations have achieved success without having to face similar challenges that other minority groups have had to face, while in reality, they have also been subject to discrimination, racism, and language barriers.

Although this paper focuses on parental help-seeking behaviours for Asian-American children, it is important to note the generational and cultural implications of the model minority myth. For instance, research has shown that second-generation Asian American young adults are particularly vulnerable to the harmful effects of the myth (12). They often grapple with the combined effects of the MMM, meeting parental expectations, and navigating discrimination and microaggressions within society. These factors contribute significantly to poor mental health outcomes, with young Asian American women reporting some of the highest suicide rates (12).

Moreover, research has shown that Asian American elders face some of the highest suicide rates and the lowest rates of professional service utilization. This could be attributed to a limited understanding of mental health and suicide within these communities, partly driven by the stereotype that Asian Americans are highly successful and do not face significant challenges (12).

Although the “model minority myth” is often viewed as a positive stereotype, research suggests that it can result in internalized racialism, which can have detrimental effects on health and help-seeking behaviors. Internalized racialism refers to the process in which a specific group internalizes stereotypes about them that are held by other groups (11). While it is widely acknowledged that many Asians do not support the “model minority” stereotype, studies have shown that internalized racialism remains a notable predictor of psychological distress (10, 11). This suggests that even those who reject this stereotype may still feel pressure to conform to its expectations, leading to feelings of inadequacy (10). Furthermore, various studies have found that falsely endorsing this stereotype can lead to a greater likelihood of not seeking help, whether for school, mental or physical needs, even though they may need it (10, 11, 13).

It is crucial to acknowledge that negative effects on help-seeking behaviors are not caused by the endorsement of this positive stereotype. The root of the issue lies in internalized racialism, which shifts the impact from the group to the individual level. The extent to which an individual internalizes the stereotype varies depending on their social, racial, and historical background. Understanding how Asians internalize this stereotype and the cultural stigma associated with seeking help, will aid clinicians in better understanding their clients and help them provide care in culturally appropriate ways (11).

In addition, there is evidence that the model minority myth may be associated with the lack of research related to mental health problems among Asian Americans. The myth perpetuates the belief that Asian Americans are less likely to experience psychological distress and therefore do not need assistance at the individual, societal, or governmental level (11).

### Protective factors

While studies have explored the factors that can negatively influence help-seeking behaviors, protective factors can serve as avenues for positive outcomes among Asian Americans and ADHD-related challenges. A comprehensive analysis was conducted to evaluate the key factors influencing Asian Americans' mental health help-seeking behaviors. The review categorized the factors based on specific subsets of Asian communities, such as Chinese, Korean, Filipino, Asian Indian, and Vietnamese. Notably, a consistent protective factor was identified across all groups, namely acculturation. Acculturation is the process of adopting the customs and ideas of another's culture, typically the one viewed as more dominant (14). Those who had spent an extended period in the United States, demonstrated a higher proficiency in English, and understood American culture and healthcare were more likely to seek help (11). This is described to be caused by a reduced amount of perceived cultural barriers (15). Acculturation, in this context, is considered a protective factor because it enables individuals to better navigate the healthcare system and tend to feel more comfortable seeking mental health support. This increased comfort level reduces perceived cultural barriers that might otherwise discourage help-seeking behaviors. Although acculturation is a protective factor, it is unfair to assume that individuals need to adjust their culture and beliefs to feel comfortable seeking care in American society. These factors further prove the need to create a culturally adjusted help-seeking model to better help meet the needs of Asian American individuals.

### Risk factors that impede help-seeking

While limited protective factors are related to help-seeking behaviors, risk factors have been identified in the literature. One commonly cited risk factor that negatively influences help-seeking behaviors among Asian American individuals is adherence to traditional and cultural values. Findings from a study that assessed cultural factors that shape help-seeking among Chinese-American parents revealed a correlation between traditional values and a reduced likelihood of seeking help for child behavior problems (16). A systematic review that looked at various subsets of Asian Americans' help-seeking behaviors found that feelings of shame, failure, family pride, and “loss of face”, were all significant barriers to seeking out formal healthcare services. “Loss of face” is defined as suffering disgrace, humiliation or embarrassment (15). Stigma, shame, cultural influence, and immigrant experience have all played significant roles in influencing help-seeking behaviors, with a noted theme of prioritizing cultural values over seeking assistance for mental health concerns. There is an emphasis on the importance of culturally competent mental health services to address the specific needs of Asian American families. Such services include cultural brokering, where providers assist in navigating cultural differences, supporting families through treatments, and utilizing cultural knowledge to tailor care effectively (17).

In addition to psychological barriers, structural barriers can have a significant impact as well. Many studies have indicated a lack of financial resources, knowledge barriers, and lack of trust in healthcare professionals as barriers to help-seeking (1, 4, 5, 15). There can be limited awareness of concepts of mental illness within Asian American communities, which can contribute to a lack of knowledge about the condition, its symptoms, and available treatments (15).

Asian Americans comprise a large heterogeneous group with different cultural norms, making it essential for clinicians to gain a deeper understanding of cultural nuances to provide culturally safe care (18). However, this can be a challenge; evidence shows that Asian Americans may perceive healthcare providers as authority figures and are, therefore more likely to tell providers what they think they want to hear to avoid raising issues (19). Providers need to be aware of this belief and take the time to reassure patients that they will not be judged and to ask patients about their cultural views, treatment expectations, and coping patterns to ensure proper care is provided (19).

## Proposed help-seeking model adjustments

Although the help-seeking model designed by Eiraldi et al. (6) is comprehensive, adjustments are needed to be more applicable to Asian American individuals' needs. Presently, the model fails to incorporate certain unique cultural perspectives that are specific to the Asian American community. Variables related to culture are assumed to influence every stage in the help-seeking model (6), therefore, the suggestions we provide are based on the premise that cultural context must be considered in every step and understood to properly use the model.

The literature demonstrates that Asian Americans possess distinctive cultural perspectives that can hinder their willingness to seek assistance (1, 4, 5, 11, 15, 16, 19). Factors such as shame, family honour, internalized racialism, and fear of social disgrace were identified as traits that impact their decision-making process. Given these insights, we propose incorporating these traits into the “decision to seek help” phase, particularly under “fears”. The research highlights that certain Asian American communities may harbor apprehension regarding the negative evaluations that psychiatric diagnoses, such as ADHD, may generate (15). It is important to consider the fear of diminished family pride, “loss of face,” and shame when understanding why Asian American parents may not seek care for their child's ADHD. These factors can significantly impact parental decisions regarding seeking treatment for their child.

We also recommend including the impact of the model minority myth under societal factors in the Service Selection stage. As previously mentioned, this myth implies that Asian Americans effortlessly adapt to American norms and achieve great success, leading many people to feel the need to conform to this notion (11). Even if individuals acknowledge a problem and opt to seek assistance, selecting the appropriate service can be difficult due to concerns about disappointing society and failing to live up to this favorable stereotype. By integrating the model minority myth into the framework, providers can gain a better understanding of the myth and the specific obstacles that hinder help-seeking behaviors among Asian Americans. Providers can modify their approach to encourage individuals to seek care. They can also provide educational resources that recognize ADHD as a prevalent condition that cannot be prevented or avoided, thereby reducing the stigma associated with not conforming to the stereotype. Recognizing society's role in perpetuating this stereotype is crucial for future research, educational initiatives, and policy-making.

Further, to strengthen the help-seeking model for Asian Americans, “practitioner-family partnerships” should be included (20). “Practitioner-family partnerships” refers to the collaborative effort between healthcare providers and families in the overall care process, ensuring that decisions regarding one's health are made jointly rather than by one party alone. Particularly in the context of Asian American communities, this partnership is a collaborative relationship between the healthcare provider and the family to enhance the quality of care and address barriers to help-seeking behaviors. By adding this under the service utilization section under quality of care, establishing collaborative relationships becomes crucial. Since Asian-American individuals perceive healthcare providers as authority figures, practitioners must actively engage with families, creating non-judgmental spaces that reassure the parents about the confidentiality of their concerns (21). This can be done by active engagement and cooperation between practitioners and families to navigate cultural nuances, mitigate stigma, and ensure effective communication and decision-making in the healthcare process.

These adjustments aim to create a more supportive and culturally sensitive pathway for Asian-American families seeking assistance with ADHD-related challenges, thereby dismantling the barriers to help-seeking behaviors within the community.

## Discussion

The current evaluation of ADHD among Asian American populations has a significant gap in research, particularly in testing and refining help-seeking models. Adapting the help-seeking model for Asian Americans with ADHD requires an understanding of diverse experiences within this population. Addressing the minority myth, acknowledging protective and risk factors, and promoting cultural competence are essential steps toward reducing treatment disparities and improving mental health outcomes for Asian Americans. The adjustments made to the help-seeking model, particularly the incorporation of practitioner-family partnerships and the recognition of the model minority myth within societal factors, offer insights into creating a more culturally sensitive help-seeking model.

Moving forward, future research should investigate and refine cultural adaptations to existing help-seeking models for ADHD. Our recommendations were derived from an analysis of mental health help-seeking behaviors. While ADHD may be a factor, the focus of these studies was primarily on symptoms of depression and anxiety. To better understand how Asian Americans seek help for ADHD, it is crucial to conduct further research that explores different subsets of this diverse population. Disaggregated data for each group within the Asian community is essential to create help-seeking models that are tailored to their specific needs. Recognizing the potential barriers to research participation, such as language, cultural stigma, and mistrust is crucial in designing an improved model that is sensitive to the needs of Asian Americans (1, 4, 5, 11, 15, 16, 19). These changes can advance research, bridging the existing gaps in ADHD diagnoses, treatments, and help-seeking behaviors within the Asian American population.

## Figures and Tables

**Figure:**
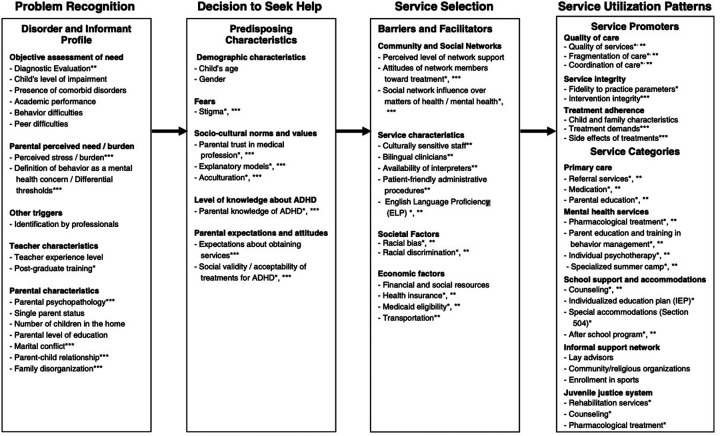
Extracted from Eiraldi, R., Mazzuca, L., Clarke, A., & Power, T. Service utilization among ethnic minority children with ADHD: A model of help-seeking behavior. Administration and Policy in Mental Health and Mental Health Services Research. 33(5): 607-622. 2006, Springer Nature.
